# BMPs Regulate *msx* Gene Expression in the Dorsal Neuroectoderm of *Drosophila* and Vertebrates by Distinct Mechanisms

**DOI:** 10.1371/journal.pgen.1004625

**Published:** 2014-09-11

**Authors:** Francisco F. Esteves, Alexander Springhorn, Erika Kague, Erika Taylor, George Pyrowolakis, Shannon Fisher, Ethan Bier

**Affiliations:** 1 Section of Cell and Developmental Biology, University of California, San Diego, La Jolla, California, United States of America; 2 Institute for Biology I, Faculty of Biology, Albert-Ludwigs-University of Freiburg, Freiburg, Germany; 3 Spemann Graduate School of Biology and Medicine (SGBM) Albert-Ludwigs-University of Freiburg, Freiburg, Germany; 4 Research Training Program GRK 1104, Albert-Ludwigs-University of Freiburg, Freiburg, Germany; 5 Department of Cell and Developmental Biology, University of Pennsylvania, Philadelphia, Pennsylvania, United States of America; 6 BIOSS Centre for Biological Signalling Studies, Albert-Ludwigs-University of Freiburg, Freiburg, Germany; University of California Davis, United States of America

## Abstract

In a broad variety of bilaterian species the trunk central nervous system (CNS) derives from three primary rows of neuroblasts. The fates of these neural progenitor cells are determined in part by three conserved transcription factors: *vnd*/*nkx2.2*, *ind*/*gsh* and *msh*/*msx* in *Drosophila melanogaster*/vertebrates, which are expressed in corresponding non-overlapping patterns along the dorsal-ventral axis. While this conserved suite of “neural identity” gene expression strongly suggests a common ancestral origin for the patterning systems, it is unclear whether the original regulatory mechanisms establishing these patterns have been similarly conserved during evolution. In *Drosophila*, genetic evidence suggests that Bone Morphogenetic Proteins (BMPs) act in a dosage-dependent fashion to repress expression of neural identity genes. BMPs also play a dose-dependent role in patterning the dorsal and lateral regions of the vertebrate CNS, however, the mechanism by which they achieve such patterning has not yet been clearly established. In this report, we examine the mechanisms by which BMPs act on cis-regulatory modules (CRMs) that control localized expression of the *Drosophila msh* and zebrafish (*Danio rerio*) *msxB* in the dorsal central nervous system (CNS). Our analysis suggests that BMPs act differently in these organisms to regulate similar patterns of gene expression in the neuroectoderm: repressing *msh* expression in *Drosophila*, while activating *msxB* expression in the zebrafish. These findings suggest that the mechanisms by which the BMP gradient patterns the dorsal neuroectoderm have reversed since the divergence of these two ancient lineages.

## Author Summary

The trunk nervous system of both vertebrates and invertebrates develops from three primary rows of neural stem cells whose fate is determined by neural identity genes expressed in an evolutionarily conserved dorso-ventral pattern. Establishment of this pattern requires a shared signaling pathway in both groups of animals. Previous studies suggested that a shared signaling pathway functions in opposite ways in vertebrates and invertebrates, despite the final patterning outcomes having remained the same. Here, we employ bioinformatics, biochemistry, and transgenic animal technology to elucidate the genetic mechanism by which this pathway can engage the same components to generate opposite instructions and yet arrive at similar outcomes in patterning of the nervous system. Our findings highlight how natural selection can act to conserve a particular output pattern despite changes during evolution in the genetic mechanisms underlying the formation of this pattern.

## Introduction

In both *Drosophila melanogaster* and vertebrates, Bone Morphogenetic Proteins (BMPs) are expressed in the epidermal ectoderm abutting the dorsal border of the neuroectoderm [1]. The genetic network that underlies formation of a centralized nervous system consisting of segregated motor and sensory centers appears to have been conserved across bilaterians (animals with right-left symmetry) [2]. BMPs are thought to exert a common function in the early epidermal ectoderm during neural induction (i.e., suppressing expression of neural genes in epidermal regions that experience peak BMP levels). BMP signaling also acts subsequently in a dose dependent fashion to pattern dorsal versus medial regions of the neuroectoderm. For example, the trunk Central Nervous System (CNS) of both invertebrates and vertebrates consists of three primary rows of neuroblasts that are determined by the expression of three conserved transcription factors. In metazoan species spanning all three primary branches (e.g., Ecdysozoa -*Drosophila*, lophotrochozoa – annelids, and deuterostomes - vertebrates) “neural identity” genes (*vnd*/*nkx2.2*, *ind*/*gsh* and *msh*/*msx*) are expressed in the same relative order and orientation with respect to the dorsal-ventral axis and an epidermal BMP source. Moreover, in a broad range of organisms, BMPs and opposing antagonists have been found to play a key role in patterning the ectoderm and establishing neuronal fates. These commonalities suggest an ancestral origin for the CNS among bilateria [1]–[4] and raise the possibility that BMPs play a conserved role in patterning the CNS axis.

Despite their consistent role in promoting epidermal over neuronal cells fates in diverse species, BMPs and other extracellular factors are deployed in diverse patterns and may act by distinct mechanisms to achieve D/V patterning [5]. For instance, in *Drosophila*, BMPs originating in the presumptive epidermis act to repress expression of neural genes during both neural induction [6] and subsequent neuroectodermal patterning [3], [7]. In vertebrates, however, the prevailing view is that BMPs act as they do in flies to repress expression of neural genes within epidermal regions early during neural induction [1], [8] but switch function later to activate expression of orthologous neural identity genes in dorsal regions of the neural tube (e.g., the *msh* orthologs *Msx1/2*) [9]. Thus, in mice, ectopic BMP signaling leads to ventral expansion of *msx* expression in the neural tube [10]. In contrast, in *Drosophila*, the absence of BMPs leads to *msh* expanding dorsally into non-neural domains [11]. In zebrafish, there is evidence that BMPs act in a bimodal fashion where intermediate BMP levels are necessary for activating *Msx* genes, while both low and high levels of BMPs repress or fail to activate these target genes [12]. Similarly, in amphioxus, a basal chordate, *msx* is expressed more broadly but at reduced levels in response to ectopic BMP signaling [13]. In Echinoderms, where BMPs and chordin are co-expressed in the ventral ectoderm that gives rise to neural tissue [14], *msx* is expressed dorsally and is activated by peak levels of BMPs that diffuse dorsally from their ventral source into non-neural regions while Chordin remains restricted to ventral regions where it blocks the BMP response in neural cells [15]. While these conserved suites of gene expression strongly suggest a common ancestral origin for BMPs in axial patterning, it is unclear whether the regulatory mechanisms establishing these patterns have been similarly conserved during evolution.

BMPs signal via hetero-tetrameric receptor complexes consisting of two type-I and two type-II subunits, which in turn phosphorylate the cytoplasmic transducing-SMAD proteins (Mothers Against Dpp (Mad) in *Drosophila*, SMAD1/5/8 in vertebrates). Once phosphorylated, pMad/pSMAD1/5/8 translocates into the nucleus in a complex with Medea/Smad4 whereupon they act as transcription factors to regulate expression of BMP target genes (reviewed in [16]). Mad and Medea (Med) bind DNA as a heteromeric complex consisting of two Mad subunits and one Med subunit to regulate genes through interactions with binding sites composed by a Mad (GC-rich) site separated, by a variable length spacer, from a Med (Smad Binding Element or SBE) site. One of the best characterized such sites in *Drosophila* is the *brinker* (*brk*) Silencer Element (SE) which has a spacer length of 5 nucleotides [17]–[20]. Brk encodes a transcriptional repressor protein and the *brk* gene itself is repressed by Dpp (the *Drosophila* BMP4 homologue) signaling. Repression of *brk* through its SEs requires the presence of the zinc-finger protein Schnurri (Shn) [21]–[23], which is provided maternally and is also expressed zygotically in dorsal epidermal regions of the early embryo. Hence, in *Drosophila*, genes that are repressed by BMPs have been found to have binding sites for pMad/Med/Shn (henceforth, pMMS) complexes in their cis-Regulatory Modules (CRMs) while genes that are directly activated by BMPs, such as the inhibitory SMAD *daughters-against-dpp* (*dad*), contain activating elements (AE) in their CRMs [24]. These AE elements also share a bipartite configuration (GC-rich/spacer/SBE), but have configurations (spacing and sequence constraints) that do not allow for Shn binding and lead instead to the recruitment of activating transcriptional co-factors.

Here, we compare BMP-mediated regulation of CRMs controlling the expression of the *Drosophila msh* and zebrafish and mouse *msx* genes in the early dorsal nerve chord. We identify zebrafish and mouse *msx* neuroectodermal CRMs that drive expression in the dorsal neuroectoderm. We find that both *Drosophila msh* and zebrafish *msxB* CRM-reporter transgenes respond to BMPs and characterize BMP responsive sites within these elements. Consistent with prior genetic studies [7], the *Drosophila msh* CRM contains *Shn*-dependent SE sites that are required for BMP repression. Surprisingly, it also harbors sites that resemble known BMP-responsive activation sites, which, however, do not bind to pMad/Medea (pMM) complexes *in vitro*, but are nonetheless required for *msh* expression. In addition, we characterize a single SMAD binding site with a novel spacing of SMAD1/5/8 and SMAD4 binding motifs in a minimal zebrafish *msxB* CRM that is required for dorsal neuroectodermal expression. This comparison suggests that while overall gene expression patterns have been conserved between flies and zebrafish and are both regulated by BMP signaling, distinct mechanisms have evolved to generate the shared output patterns in these two widely separated metazoan lineages.

## Results

### The *Drosophila msh* CRM responds to BMPs

**Figure 1:**
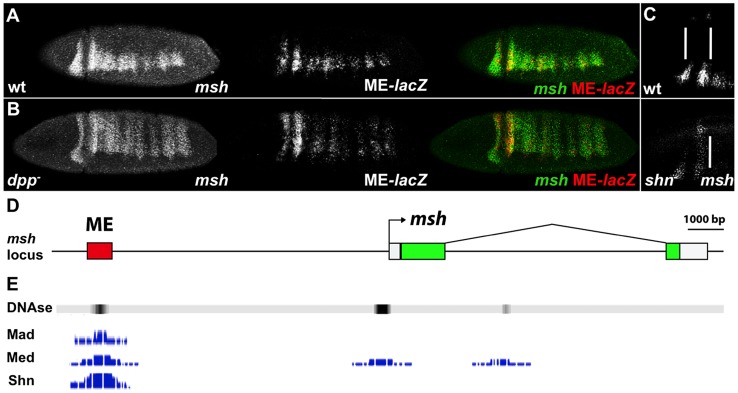
The *msh* CRM responds to BMPs. Lateral and dorso-lateral views (anterior to the left) of blastoderm stage *Drosophila* embryos showing *msh*, *shn* and ME-*lacZ* reporter construct expression detected by *in situ* hybridization, and schematic overview of the *msh* locus. (A) Transgenic embryos carrying the *lacZ* reporter gene under control of the ME were hybridized with probes against *msh* (green) and ME-*lacZ* (red). (B) The ME-*lacZ* construct was crossed into a *dpp* null background. Expression of both *msh* and ME-*lacZ* expands dorsally in *dpp*- homozygous mutant embryos. (C) In *shn*- homozygous zygotic mutant embryos, anterior *msh* expression expands towards the dorsal regions of the embryo compared to wild-type. A maternal contribution of *shn* remains intact in these embryos. (D) The *msh* locus depicting the location of the ME CRM. (E) Genome wide ChIP data representation depicting peaks of Mad, Med and Shn binding signal as well as DNase hypersensitivity sites reflecting regions of open chromatin in the ME CRM region.

A 700 bp *msh* CRM (henceforth referred to as ME for Msh Element) has been identified that is directly repressed by Ind [25]. The response of the ME to BMP-mediated regulation has not yet been investigated, however. As is the case for the endogenous *msh* gene (Fig. 1A), the expression of a *ME-lacZ* construct expands throughout the dorsal region of the embryo in *dpp*- mutants (Fig. 1B). In order to determine whether Dpp regulates *msh* directly or indirectly, we analyzed BMP regulation of the ME element. Consistent with a direct role of BMP signaling on this CRM, genome wide chromatin immune precipitation (ChIP) data [26], [27] revealed DNA binding sites for the BMP effectors Mad, Medea and Shn within the ME region in blastoderm stage embryos (available on the UCSC genome browser - http://genome.ucsc.edu/ or the Berkeley Drosophila Transcription network Project - http://bdtnp.lbl.gov/Fly-Net/chipchip.jsp) (Fig. 1D). We confirmed the involvement of Shn in regulating *msh* within the neuroectoderm by examining homozygous zygotic *shn*- mutant embryos, which exhibit a partial dorsal expansion of *msh* expression (Fig. 1C).

### The *msh* CRM contains direct BMP-responsive sites involved in repression

To identify BMP responsive sites within the ME, we first scanned this element for known consensus binding sites for Mad, Med, Shn, and Brk. The two best characterized BMP responsive elements are the Silencer Element (SE), which binds a trimeric complex comprised of pMMS (GNCGNC(N)_5_GNCTG), and the activator element (AE), which binds pMM heteromers (GGCGCCA(N)_4_GNCV). Brk binding sites ((T)GGCGYY) overlap with a subset of AE elements [24]. Although there are no perfect consensus SE, AE, or Brk sites within the ME, we identified several candidate sites with either single base-pair mismatches to the SE or AE elements or variable spacer length (N)_5–6_. We defined three such candidate SE sites (SE1, SE2 and SE3) with a single nucleotide mismatch and two conserved candidate AE sites with a spacer of 6 nucleotides (conforming to the expanded consensus: GNCGNC(N)_6_GNCV) and tested each of these sites for direct DNA binding of pMM or pMMS complexes *in vitro* using Electrophoretic Mobility Shift Assays (EMSAs).

**Figure 2:**
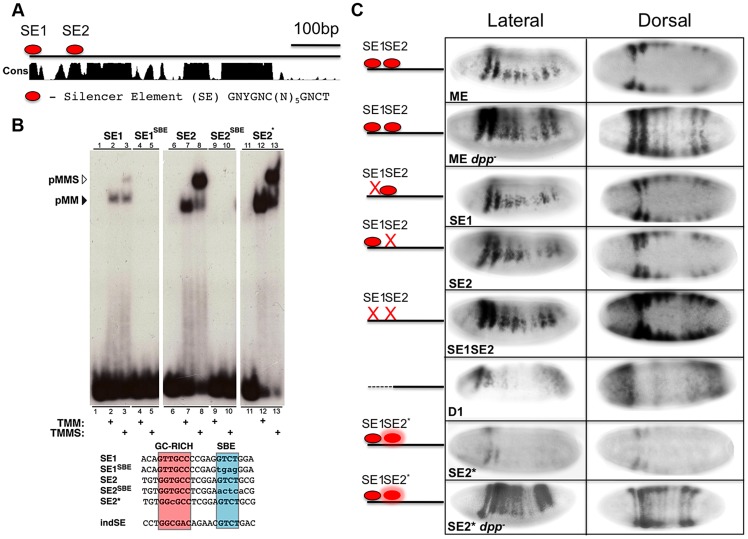
The *msh* CRM contains BMP Silencer Elements contributing to dorsal repression. (A) Diagram of ME showing the location of Silencer Elements SE1 and SE2 within highly conserved regions. Conservation score (Cons) is based on alignment of the ME region in 12 *Drosophila* species [61]. (B) Autoradiograph of electrophoretic mobility shift assay gels with radiolabeled oligonucleotide probes incubated with extracts from S2 cells over-expressing activated Tkv (to induce BMP signaling), Med and Mad in the presence or absence of over-expressed C-terminal Schnurri (Shn). EMSA probe sequences depict the location of the GC-rich (red) and SBE (blue) motifs as well as the *ind* SE site [20]. Mutations relative to wild type sequences are indicated by lower case. SE1 and SE2 containing probes show higher molecular weight retardation when Shn is present versus Med and Mad alone (lanes 1–3 and 6–8). When the SBE motif of either SE1 or SE2 is mutated, probe retardation is no longer observed (lanes 4/5 and 9/10). When SE2 is mutated to conform to the canonical SE consensus, SE2*, the amount of probe retained appears higher compared to wild-type SE2 (lanes 11–13, see Fig. S1D for further verification of this effect in a competition assay). (C) Lateral and dorsal views (anterior to the left) of *in situ* hybridization detection of *lacZ* expression in ME-*lacZ* embryos demonstrating the *in vivo* effects of mutating SE1 and SE2 SBE motifs. ME: wild type embryos containing the intact ME-lacZ construct; *dpp*: *dpp*- mutant embryos show complete dorsal expansion of *lacZ* expression; D1: deletion of the first 100 bp of ME spanning the SE1 SE2 region leads to partial dorsal expansion accompanied by reduced levels of expression; SE1: mutating SE1 leads to modest dorsal expansion; SE2: mutating SE2 leads to significant dorsal expansion; SE1 SE2: mutating both SE1 and SE2 results in yet more pronounced dorsal expansion although not as extensive as observed for the wt-ME in a *dpp*- mutant background; SE2*: Converting SE2 to an optimal (*ind*-like) SE site results in reduced *lacZ* expression; SE2* *dpp*-: SE2* in a *dpp*- background shows complete dorsal expansion of *lacZ*.

The SE1 and SE2 candidate silencer sites (Fig. 2A) both conform to the relaxed consensus of GNYGNC(N)_5_GNCTG (where Y can be either C or T). EMSA experiments using DNA oligonucleotide probes reveal that pMM and pMMS complexes assembled on the SE1 and SE2 sites in a BMP dependent fashion (Fig. 2B) but not on the SE3 site (Fig. S1C). As expected, mutation of the Med (SBE) motif within the SE1 (SE1^SBE^) or SE2 (SE2^SBE^) sites abolished binding of all BMP responsive complexes *in vitro*. In contrast, none of the candidate AE or Brk sites bound pMM, pMMS, or Brk complexes (Fig. S1C) (see below however, regarding effects of mutating or deleting the candidate AE sites).

In order to test the *in vivo* roles of the SE sites, we mutated each site (i.e., using the same SBE mutations that abolished all BMP responsive DNA binding *in vitro* described above) and generated a series of small deletions spanning virtually the entire ME (i.e., all but 36 bp). These mutant constructs were inserted into the same chromosomal integration site as the reference ME construct using the PhiC31 transgenesis system [28]. Deletion of the 5′ most 100 bp of ME, which contains both SE sites, led to dorsal expansion of reporter gene expression (Fig. 2C). Transgene expression, however, was also weaker within its normal neuroectodermal domain, suggesting that contributing activation sites are also present within this region. Targeted mutation of the individual SE1 and SE2 sites also led to discernable dorsal expansion of reporter gene expression, which was more pronounced for the SE2 mutant. Mutating both SE sites in combination (SE1, SE2 double mutant) resulted in more prominent dorsal expansion than observed for either mutant alone, but still less than that observed for the wild-type ME (or the endogenous *msh* gene) crossed into a *dpp*- mutant background. We conclude that SE elements mediate direct BMP-dependent repression of the ME and that additional direct or indirect BMP-dependent inputs also contribute to negatively regulating this CRM.

### Differential affinities of pMMS complexes for SE sites in the *ind* and *msh* CRMs may contribute to threshold-dependent repression of these genes

Our prior genetic studies revealed that BMP signaling is more effective in repressing expression of *ind* than *msh* [7]. One possible explanation for this differential response is that the *ind* CRM might contain higher affinity SE sites than those in the *msh* CRM. Indeed, a single perfect consensus matching SE site in the *ind* CRM (Fig. 2B) has been shown to be required for repression of this element dorsally [20], [29]. In line with the possibility that SE sites in the *ind* and *msh* CRMs have differing affinities for binding pMMS complexes, modifying the SE2 site by one base-pair to adhere to the optimal SE consensus resulted in greater pMMS binding (Fig. 2B - SE2*), which was most evident in competition experiments (Fig. S1D). We tested whether the optimized *ind*-like SE2* site would result in repression of *msh* CRM activity *in vivo*. In support of this site being more effective at recruiting repressive pMMS complexes, reporter gene expression driven by the SE2* ME was greatly reduced relative to that of the wild-type ME. This reduced expression was BMP-dependent since SE2*ME-driven reporter gene expression was restored and expanded throughout the dorsal region in a *dpp*- mutant background to a degree comparable to that observed for the intact ME (Fig. 2C). Taken together, these results suggest that differential affinities of pMMS complexes for SE sites in the *ind* and *msh* CRMs contribute to the mechanism by which silencer elements mediate graded BMP responses of these two genes in the *Drosophila* neuroectoderm.

### The *msh* CRM contains essential activation sites that resemble BMP responsive sequences

**Figure 3:**
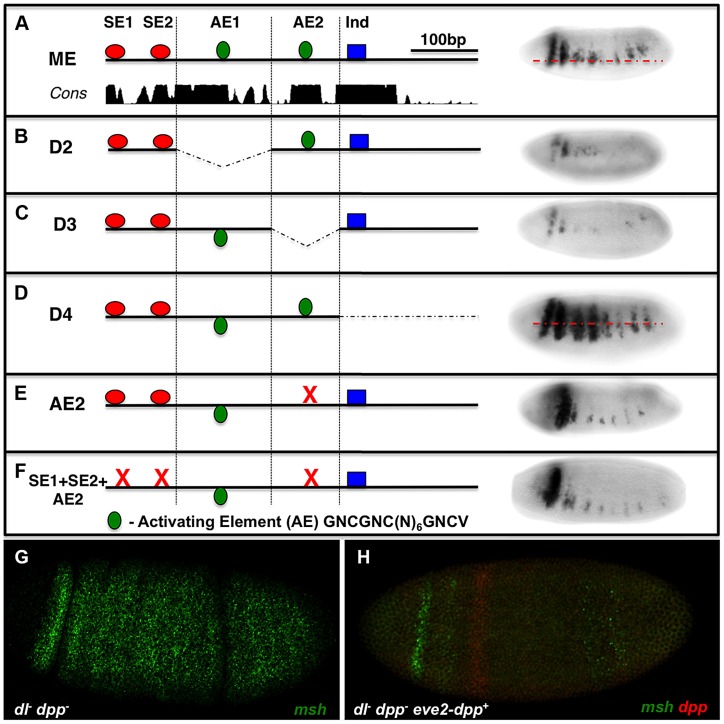
The *msh* CRM contains AE-resembling sites required for expression. Embryos are viewed laterally (anterior to the left). ME deletions spanning 4 sequential blocks (D1-D4) contain distinct islands of conserved sequences on sequence conservation and binding site clusters. *lacZ*-reporter constructs carrying these deletions were then tested *in vivo* in transgenic embryos to identify additional potential BMP responsive sequences. (A) Wild-type ME depicting the location of putative AE sites in relation to SE sites and an Ind binding site. (B) Deletion of D2 where AE1 is located results in reduced *lacZ* reporter expression. (C) Deletion of D3, which contains the AE2 region results in severely reduced *lacZ* reporter expression. (D) Deletion of D4 results in ventrally expanded *lacZ* reporter expression consistent with an essential Ind repressor binding site being present [7]. (E) Site-directed mutagenesis of AE2 results in similarly reduced reporter expression as observed for the D3 deletion, demonstrating an essential role of AE2 site as an activation sequence. (F) A triple mutant comprised of point mutations in the SE1, SE2 and AE2 sites results in reduced reporter expression comparable to that observed in the AE2 mutant alone suggesting that this site does not mediate BMP-dependent activation. The AE consensus shown (GNCGNC(N)_6_GNCV) is an expanded version based on the standard consensus indicated in the text (GGCGCCA(N)_4_GNCV) and on our hand curation from the literature. In addition, sensitized embryos were tested for potential *msh* activation by particular doses of Dpp. (G) *msh* expression in an embryo lacking maternal Dorsal and lacking zygotic Dpp (*dl- dpp-*), *msh* (green) is ubiquitously expressed as previously reported [11]. (H) The addition of a single copy of a wild-type *dpp* gene under the control of the *even-skipped* stripe 2 CRM, which creates a Dpp gradient emanating from the zone of expression (red) [7] in *dl- dpp*- embryos abolishes most *msh* expression (green) throughout the embryo. These results suggest that Dpp does not have a positive role in *msh* regulation in the absence of Dorsal signaling in *Drosophila* at any dose.

As mentioned above, in our initial search for BMP-responsive sites in the ME we identified two sites that were similar to activation elements (AE) but that did not bind pMM complexes in EMSA assays (Fig. S1C). We nonetheless tested for potential roles of these sites by deleting them or creating a point mutation in one of them (AE2). Deletions encompassing either AE1 or AE2 or the AE2 point mutation greatly reduced ME-lacZ expression (Fig. 3B,C,E), while deletion of the 3′ most region containing a previously reported Ind site [25] resulted in ventral expansion of reporter gene expression as expected. We tested the possibility that activation of the ME via AE2 might be balanced against repression mediated by the SE1 and SE2 sites by constructing a triple mutant in which all three sites were eliminated. We reasoned that if the AE2 site, which acts as a bonafide activation site, functions in a BMP independent manner, combining it with the double SE site mutant might result in loss of expression (the AE mutant phenotype). On the other hand, if the AE2 site were providing an important activation function in the neuroectoderm via BMP signaling, the triple mutant should at least show ectopic expression dorsally (e.g., if this was a BMP-dependent activation site, relieving repression would give rise to normalized expression since we would be removing both activating and repressing components). We found loss of expression in this triple mutant comparable to that of the AE2 single mutant (Fig. 3F), suggesting that activation via the AE2 site is BMP-independent.

Although the above analysis suggests that the AE2 site acts in a BMP-independent fashion, we further examined the possibility that BMPs might play an activating as well as repressive role in regulating *msh* expression. Embryos that are *dorsal*- (maternal); *dpp*- (zygotic) double mutants express *msh* ubiquitously [11]. To test whether there might be a threshold at which Dpp enhances rather than suppress *msh* expression, we attempted to augment *msh* expression locally by generating embryos that lack Dorsal and whose only source of Dpp is one copy of *dpp* driven in a narrow stripe by the *eve* 2 CRM (Fig. 3G,H) or by, adding progressive amounts of Dpp (by varying copy number of the *dpp* locus – Fig. S2A,B). In both cases, we observed only a diminution in *msh* expression, further arguing against any activating role for Dpp. Finally, we considered the possibility that BMPs might act indirectly to regulate *msh* expression via non-canonical mechanisms (e.g. via ETS or the HMG-box Cic transcription factors) by altering EGF-R signaling. We found no evidence, however, for a role of EGFR signaling in influencing the position of the dorsal border of *msh* expression (Fig. S2C-E). In aggregate, our experiments suggest that BMP-dependent regulation of the ME is mediated by SE sites and by additional inhibitory inputs, which may act either directly or indirectly.

### Identification of early embryonic vertebrate *msx* CRMs

**Figure 4:**
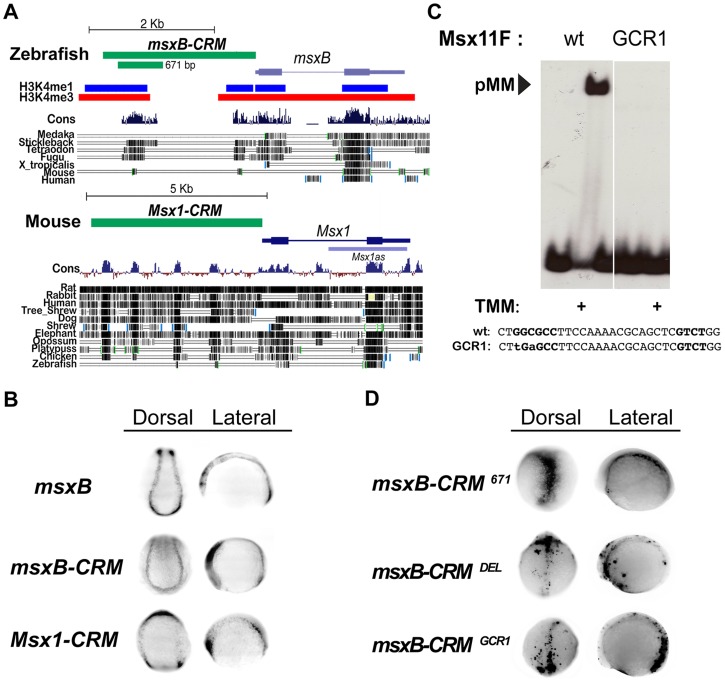
Identification of vertebrate *msx* CRMs. (A) Representation of the zebrafish *msxB* and mouse *Msx1* loci depicting the location of the CRMs and vertebrate sequence conservation (Cons) (http://genome.ucsc.edu/). For zebrafish, histone 3 lysine 4 single and triple methylation patterns indicative of open chromatin are also indicated. Block conservation tracks for select species are represented for both loci. (B) *In situ* expression driven by *msx* CRMs. Dorsal (anterior to the top) and lateral (anterior to the left) views of transgenic zebrafish embryos at the open neural plate stages (3–6 somites). Embryos were injected with either *msxB*-CRM or *Msx1*-CRM constructs driving *gfp* and stable transgenic lines were subsequently bred. Stable transgenic embryos were stained for *msxB* and *gfp* expression, which was detected by *in situ* hybridization. Both CRMs drive patterns resembling the endogenous *msxB* pattern. The zebrafish DNA isolated contains sufficient information to drive a pattern resembling the endogenous *msxB* expression pattern. The cloned mouse CRM also is capable of responding to regulatory cues in the zebrafish embryo to drive expression resembling that of the zebrafish *msxB* gene (as well as the endogenous *Msx1* gene in mice [62]). Note that this embryo is tilted in a slightly more rostral direction than the other embryos shown from the dorsal perspective, which results in bands of anterior expression coming into view. (C) EMSA experiment in which a radiolabeled oligonucleotide probe carrying the zAE element (Msx11F in Fig. S3) was incubated with extracts from S2 cells over-expressing activated Tkv (to induce BMP signaling), Med and Mad. When the GC-rich region of the mad1 binding site is mutated (GCR1), pMM biding is abolished (the same loss of binding was also observed for the mutation in the mad2 site, GCR2 - see Fig. S3C). (D) Mutation of the pMM zAE site greatly reduces specific expression driven by the 671 bp *msxB* CRM. Dorsal (anterior to the top) and lateral (anterior to the left) views of injected zebrafish embryos (6–8 somites). Embryos were injected with GFP-reporter constructs under the control of the intact 671 bp *msxB*-CRM, a 30 bp mutant deleting the zAE (*msxB*-CRM^DEL^), or a point mutant version of the zAE that abolishes pMM binding – see panel C (*msxB*-CRM^GCR1^). Both zAE mutant constructs show greatly reduced reporter expression. Transient *gfp* mRNA expression was detected by *in situ* hybridization.

The above analysis of the *msh* CRM in *Drosophila* is consistent with genetic data indicating that BMPs act by dosage sensitive repression of neural identity gene expression [7]. To determine the mechanism by which BMPs regulate expression of orthologous vertebrate *Msx* genes we sought to identify the zebrafish (*Danio rerio*) *msxB* CRM using the powerful *tol2* transgenesis system [30]. We choose to focus on regulation of the *msxB* gene among the zebrafish *Msx* paralogs as this gene has the earliest onset and most specific pattern of expression in the dorsal neuroectoderm [31]. We identified a 2.4 Kb region of DNA immediately upstream of the zebrafish *msxB* coding region that drives faithful reporter gene expression in the dorsal neuroectoderm in both neural plate and early neural tube stages (i.e., 3–6 somite stage embryos) of a stable transformant line (Fig. 4A,B). This fragment has two peaks of strong sequence conservation among vertebrates, which overlap regions of predicted open chromatin [32] (Fig. 4A). Later during neural tube stages, the early neural plate expression pattern fuses into a single dorsal zone (e.g., top panels in Fig. 4D).

We also tested a 5 Kb genomic fragment upstream of the mouse (*Mus musculus*) *Msx1* gene, which like the zebrafish *msxB* CRM carries sequences lying immediately upstream of the transcriptional start site (Fig. 4A). When the mouse CRM-GFP construct was introduced into zebrafish embryos, it drove expression in a pattern (Fig. 4B) very similar to that of the fish *msxB* gene as well as that observed endogenously in mice. These results suggest that both the zebrafish and mouse CRMs contain sufficient information to correctly direct expression to the dorsal ectoderm despite the fact that they show only limited sequence conservation. These observations provide another clear example of the highly conserved function of vertebrate CRMs from lineages that diverged over 400 MYA in the absence of obvious sequence conservation in these non-coding regions [33], [34].

### The zebrafish *msxB* CRM contains a BMP responsive site mediating activation

We pared down the zebrafish *msxB* CRM in transient transformant embryos and identified a minimal 671 bp fragment containing the most conserved island that also faithfully recapitulates *msxB* expression in dorsal neuroectodermal/neural crest progenitor cells (Fig. 4D). Paralleling our approach in *Drosophila*, we searched for BMP responsive sites within the minimal *msxB* CRM by first scanning bioinformatically for candidate SE or AE sites using the SMAD1/5/8 consensus GNCKNC and SMAD4 consensus GNC(T/V) with relaxed spacing constraints, and then testing by EMSA whether oligonucleotides containing these sites could indeed assemble *Drosophila* pMM and/or pMMS complexes in response to BMP signaling *in vitro* (Fig. S3). This analysis identified a single highly conserved site (zAE) to which BMP signal-dependent pMM (but no pMMS) DNA binding was observed. The zAE contains candidate SMAD1/5/8 and SMAD4 binding sites separated by an unusually long 16 bp spacer (Fig. S3A,B). These sites are also present in mouse albeit with different spacing (12 bp). Further analysis of this binding motif revealed that the SMAD1/5/8 and SMAD4 sites are each required, suggesting that the functional zAE includes both sites (Fig. S3C). Changing the sequence or length of the spacer DNA linking the two sites did not affect the ability to form pMM complexes *in vitro* indicating that the exceptional length of the zAE spacer is not required for SMAD complex formation *in vitro*. Interestingly, however, changing the linker length to 5 bp allowed the formation of trimeric pMMS complexes (Fig. S3D).

We generated a 36 base pair deletion spanning the zAE (and both the SMAD1/5/8 and SMAD4 candidate binding sites – DEL mutant) in the context of the 671 bp *msxB* CRM and observed that GFP reporter gene expression was lost in transient transformant embryos (Fig. 4D). Similarly, mutation of two core base pairs in the GC-rich region of the zAE (GCR1 mutant), which abolished pMM binding *in vitro* (Fig. 4C; Fig. S3C), also reduced reporter expression *in vivo* in transient transformant embryos (Fig. 4D). These results indicate that a single BMP responsive site within the 671 bp zebrafish *msxB* CRM is required for mediating reporter gene activation by this element *in vivo*.

### Fly and vertebrate *msx* CRMs respond oppositely to BMPs

**Figure 5:**
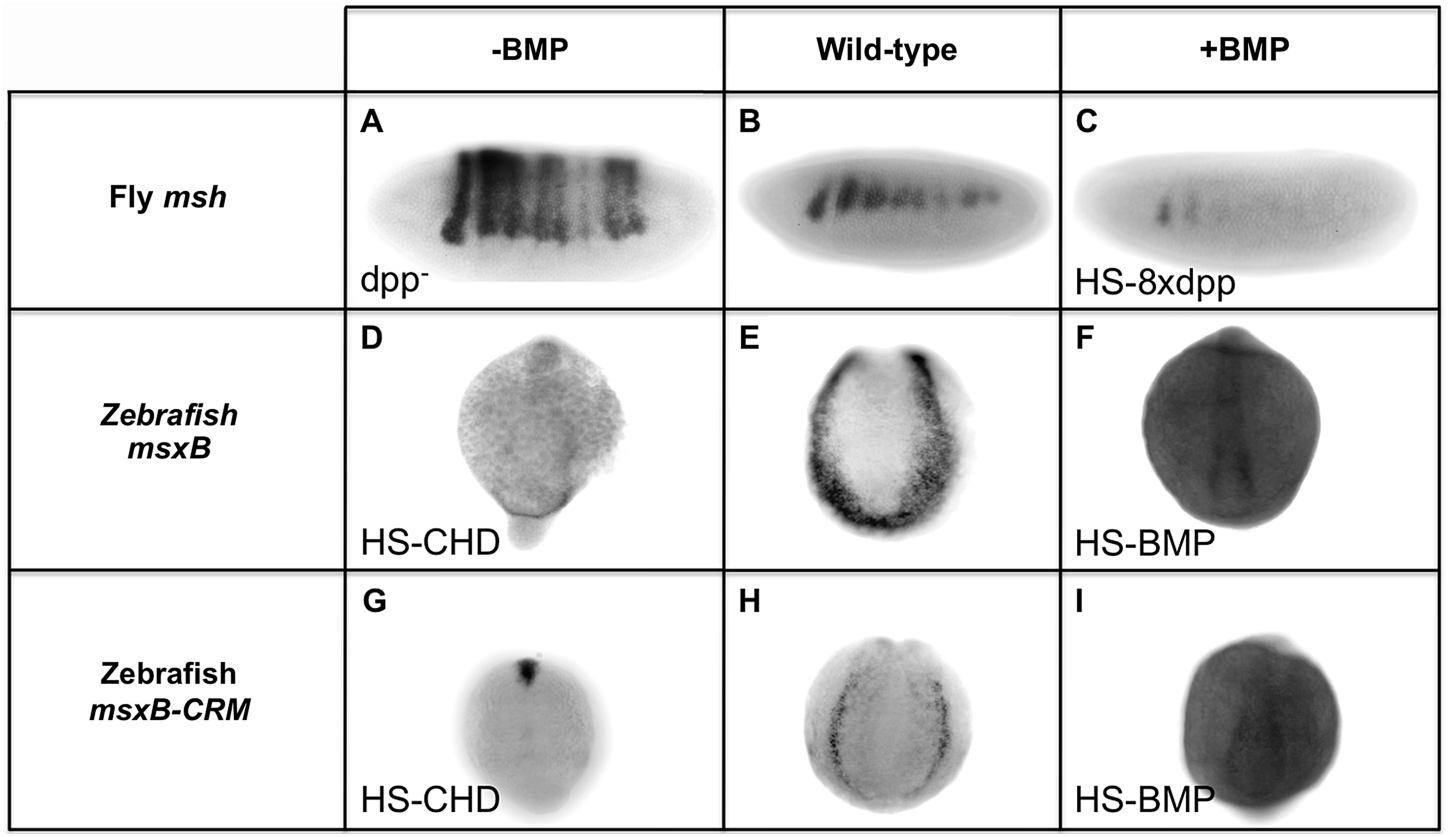
Manipulating BMP signaling elicits opposite responses from *msh* and *msxB* in *Drosophila* and zebrafish embryos. Comparison of equivalent BMP manipulations in *Drosophila* (*Dmel*) and zebrafish (*Drer*) embryos and their effects on *msh*, *msxB* and *msxB*-CRM driving GFP assayed by *in situ* hybridization. All embryos are oriented with dorsal at the top. *Drosophila* embryos are oriented with anterior to the left, while zebrafish embryos are view from a posterior perspective. Loss of BMP signaling is *Drosophila* was examined in *dpp* null mutant (*dpp* ^−^) embryos (A) and ectopic BMP signaling was generated by heat induction of transgenic embryos carrying eight copies of a heat-shock *dpp* construct (HS-8x*dpp*) (C). In zebrafish, BMP signaling was reduced by heat induction of transgenic embryos carrying a Heat Shock Chordin construct (*HS-CHD*) (D, G), while BMP over-expression was accomplished by induction of transgenic embryos carrying a Heat Shock BMP2 construct (*HS-BMP*) (F,I). In *dpp* ^−^ embryos, *msh* expression expands dorsally as shown also in Fig. 1B. In, contrast, in *HS-CHD* embryos, expression is weakened relative to the wild-type pattern for both the endogenous *msxB* gene (E) and *msxB*-CRM-*gfp* reporter construct (H). *Drosophila* HS-8x*dpp* embryos show reduced *msh* expression compared to wild type embryos (B) consistent with Dpp having a repressive action on *msh* expression, while zebrafish HS-BMP embryos exhibit ectopic expression of both the endogenous *msxB* gene and the GFP reporter gene driven by the *msxB*-CRM when compared to wild-type.

The above dissection of BMP-responsive sequences within the *Drosophila* and zebrafish *msh*/*msx* CRMs suggests that they are under opposing forms of BMP regulation: repression in *Drosophila* versus activation in zebrafish. To test this hypothesis further, we compared the response of these CRMs to alterations in BMP signaling *in vivo* (Fig. 5). In *Drosophila*, we examined *msh* and ME reporter gene expression in both a *dpp*- mutant background and in embryos ectopically expressing *dpp* in the dorsal epidermis. As mentioned above, *msh* and ME-reporter gene expression both expand dorsally in *dpp*- mutant (Fig. 1B; Fig. 5A). Conversely, ectopic *dpp* expressed from a Heat Shock-*dpp* construct (HS-*dpp*) resulted in loss of *msh* expression within its normal domain (Fig. 5C). In zebrafish, a stable transgenic line carrying the 2.4 kb *msxB*-GFP reporter construct was crossed to lines carrying either a Heat Shock-chordin (HS-CHD) or a Heat Shock-BMP (HS-BMP) construct. When the BMP antagonist Chordin was induced by heat treatment (Fig. 5G), *msxB*-GFP reporter expression was strongly suppressed, as was endogenous *msxB* expression (Fig. 5D). The opposite effect was observed in HS-BMP embryos, however, where expression of endogenous (Fig. 5F) and reporter (Fig. 5I) genes was broadened compared to control embryos (Fig. 5E and 5H, respectively) that were subjected to the same conditions. Thus, consistent with the inverse effects of mutagenizing BMP-responsive sites in the *Drosophila msh* and zebrafish *msxB* CRMs, these two elements respond in an opposing fashion to equivalent manipulations of BMP signaling *in vivo*. Our analysis strongly suggests that BMPs pattern the neuroectoderm primarily via repression in *Drosophila*, while in zebrafish, BMPs function, at least in part, to activate the orthologous *msxB* gene.

## Discussion

BMPs play a highly conserved role in neural induction and also contribute to establishment of dorsal-ventral polarity within the CNS. In the latter case, however, it has not been established whether they act through common or distinct mechanisms to effect dose-dependent patterning of neural identity genes. Since BMPs regulate expression of highly conserved members of the ancient *msh/msx* family in the dorsal neuroectoderm of both *Drosophila* and vertebrate embryos, comparing cis-regulation of these genes by BMPs provides an excellent paradigm for addressing whether cis-regulatory processes are maintained across distant taxa. Our analysis of the *Drosophila msh* embryonic CRM suggests that BMPs act in part through two SE-type binding sites that mediate repression, while activation sites do not appear to mediate responses to BMP signaling. In contrast, we identified a single SMAD binding site within an embryonic zebrafish *msxB* CRM that is required for BMP-dependent activation. These findings suggest that BMPs act on *msh*/*msx* CRMs by opposite mechanisms in these two lineages, while nonetheless driving similar output expression patterns in the dorsal neuroectoderm.

### Silencer sites mediate graded BMP responses in the *Drosophila* neuroectoderm

Mutational analysis of the *Drosophila msh* CRM in this study supports a direct role for BMP repression acting via the SE1 and SE2 sites to suppress activity of this element in the dorsal ectoderm where there are likely to be moderate levels of BMP signaling. Mutation of either of these sites results in modest dorsal expansion of reporter gene expression while elimination of both sites by point mutations or a deletion spanning both sites causes more pronounced ectopic dorsal expression. The dorsal expansion in SE1, SE2 double mutants is less complete, however, than that observed when the intact ME is crossed into a *dpp*- background, indicating that additional inputs are also involved in repressing the activity of this element dorsally. These additional BMP-dependent inputs might act either directly or indirectly. Since each of the three deletions spanning the remaining portions of the CRM (i.e. sequences outside of the deletion covering the SE1 and SE2 sites) result in reduced CRM activity it is possible that the effects of such hypothetical additional BMP responsive sites are canceled out by the deletion of necessary adjacent activation sites (e.g., deletion of the A2 site in the D3 region, Fig. 3C). If these hypothetical repressor sites act directly on the *msh* CRM they would presumably bind Mad, Medea and Schnurri, MAPK pathway transcriptional effectors, or possibly yet unknown BMP mediators alone or in conjunction with other transacting factors. Our detailed bioinformatic analysis and systematic experimental EMSA surveys have failed to identify any such sites, however. It is also possible that part of the BMP response of the *msh* CRM is mediated indirectly. For example, we have previously reported that localized overexpression of Brk can de-repress *msh* expression dorsally [7], yet there are no consensus Brk sites in the ME and we were unable to detect Brk protein binding to any closely related candidate Brk sites by EMSA (Fig. S1). Thus, Brk may act via regulating expression of other components required for BMP signaling such as the BMP type-1 receptor Thick veins [35]. Alternatively, activators of the ME may be under negative BMP/Brk regulation.

The SE1 and SE2 sites that play a role in repressing ME activity dorsally are imperfect matches to the consensus SE sites determined by Pyrowolakis and colleagues [36]. The *ind* CRM, however, which according to genetic data is more sensitive to BMP repression than *msh* [7], contains a perfect SE site required for repressing activity of this element dorsally [20]. When the SE2 site in the ME was mutated to similarly match the ideal SE consensus sequence (SE2*) it repressed ME expression in its normal dorsal ectodermal domain in a *dpp*-dependent fashion (Fig. 2C). In addition, competition experiments indicate that Mad/Schnurri/Medea bind to the *ind*-like SE element with higher affinity than the *msh* SE2 element (Fig. S1D). These combined findings suggest that differences in affinity of SE sites for forming Mad/Med/Shn complexes contribute to the distinct responses of the two CRMs to BMP-mediated threshold-dependent repression.

### A single BMP responsive site is required for activity of the *msxB* CRM

Using a combination of bioinformatics and efficient transgenesis in zebrafish we identified genomic fragments upstream of the zebrafish *msxB* and mouse *Msx1* genes that drive neuroectodermal GFP-reporter gene expression at the open neural plate stage in zebrafish embryos. Further analysis of a minimal 671 bp zebrafish CRM identified a single conserved SMAD binding site that is required for activity of this element. An novel feature of this BMP-activation site is that the SMAD1/5/8 and SMAD4 binding site motifs are separated by a 16 bp spacer, which interposes approximately one and half turns of the DNA helix between these two sites, thus differing from other characterized vertebrate BMP activation sites in which these SMAD binding sites are closer [37]. Interestingly, deletion of 11 bp (about one turn of the helix) endows this modified site with the ability to bind the pMMS repressor *complex in vitro*. Whether this unique architecture of the *msxB* BMP activation site is relevant to activity within the neuroectoderm remains to be explored.

We also examined the *in vivo* response of the endogenous zebrafish *msxB* gene and the *msxB*-CRM to inhibition of BMP signaling or ectopic expression of BMPs and compared these responses to equivalent manipulations of BMP signaling in *Drosophila*. In *Drosophila*, *msh* or ME-*lacZ* expression expands dorsally in a *dpp*- mutant while *msh* expression is repressed within its normal dorsal neuroectodermal domain by ectopic *dpp* expression. In contrast, expression of the zebrafish *msxB* gene, which is mirrored by activity of the *msxB*-CRM, is lost upon inhibition of BMP signaling and expanded or elevated in response to ectopic BMPs. Thus, both mutational analysis and *in vivo* testing suggest opposing mechanisms for BMP-dependent regulation of the *msh* and *msxB* genes in the early neuroectoderm.

Given the opposing mechanisms by which the *msh* and *msxB* CRMs respond to BMPs, it is intriguing that a site within the *msh* CRM closely resembling an activation site (AE2) is required for activation of this CRM. Also, another AE-like site (AE1) lies within a region which when deleted greatly reduces ME driven reporter gene expression, although the role of that AE1 site remains to be examined. These AE-like sites, while having only single mismatches to consensus Mad-Medea binding sites, did not bind Mad-Medea complexes *in vitro*, indicating that they are most likely not involved in mediating a BMP response. Additionally, experiments designed to identify potential positive roles of BMP signaling in regulating ME activity provided no evidence for such an effect. Given the known role of AE sites in other genes to BMP-dependent activation and the evidence that BMPs can act positively to promote *msx* gene expression in vertebrates, it is tempting to speculate that these sites could once have been BMP responsive activation sites and were subsequently co-opted by different transcription factors (possibly a TAGteam motif [38] binding protein) in the course of evolution to maintain *msh* expression in a BMP-independent fashion. Identifying such transcriptional activators is an interesting goal for future experiments.

### Evolution of conserved gene expression patterns

In *Drosophila*, Evo/Devo studies of the *even-skipped* stripe 2 CRM have suggested that regulatory mechanisms that lead to a particular gene expression pattern are extremely flexible, i.e., the same pattern can be achieved in multiple ways [39]. Accordingly, in the current case of BMP-dependent regulation of *msh*/*msxB* expression, natural selection may have operated similarly to maintain relevant gene expression patterns that fulfill a particular function (i.e. dorsal neuroectodermal expression) while allowing the upstream mechanisms generating that pattern to change over time.

As summarized above, our analysis strongly suggests that BMPs pattern the neuroectoderm primarily via repression in *Drosophila*, while in zebrafish, BMPs function, at least in part, to activate the orthologous *msxB* gene. Genetic studies and exogenous BMP treatment in zebrafish suggest that *msx* gene expression may also be repressed by high levels of BMP signaling. Whether the BMP-responsive site in the 671 bp *msxB* CRM together with other potential BMP-responsive elements mediate such a biphasic response will be interesting to address in future experiments. In the future, it will also be important to determine whether expression of other *msx* paralogs in the dorsal CNS of zebrafish (e.g., *msxC,E* [31]) or *msx* genes in other vertebrates (e.g., the murine *Msx1* neuroectodermal CRM identified here) are similarly regulated by BMPs. Analysis of these additional vertebrate *msx* CRMs should reveal whether distinct evolutionary trajectories have shaped the BMP responsiveness of these elements. Such comparative studies may also shed light on whether there is a single or multiple independent origin(s) of BMP regulation of vertebrate *msx* genes. Furthermore, analysis of the CRM driving BMP-dependent expression of an echinoderm *Msx* homolog in regions of peak BMP activity [14] will be informative since this gene is expressed in the non-neural ectoderm. In this case, one might predict finding only positively acting AE-like BMP-responsive sites.

There are two possible explanations for distinct mechanisms of BMP-regulation of *msh/msxB* expression in flies versus fish. One is that these genes independently evolved BMP responsiveness. Alternatively, BMP-dependent regulation may be an ancestral trait dating back to the first bilaterians with a condensed CNS. We favor the latter alternative for the following reasons. First, the co-linearity of *msh-msx*, *ind-gsh/pax*, and *vnd-Nkx2.2* genes relative to the source of BMPs and the BMP responsiveness of these genes in species from all three primary branches of bilateria - flies (ecdysozoa), vertebrates (deuterostome chordates), and annelid worms [40] (lophotrochozoa) - provides a compelling argument for this arrangement reflecting the ancestral state. Second, a polarized source of BMPs was present in diploblasts (e.g., corals [41], [42], jellyfish [43], and the sea anemone [44], [45]) and therefore preceded evolution of bilaterian triploblasts and a condensed CNS. Thus, it is plausible that a single species evolved a condensed CNS which deployed neural identity genes along the DV axis in much the same way that Hox genes are expressed in sequential order along the AP axis. Finally, if one looks more broadly among the 30 bilaterian phyla, a striking trend is that at least some clades within most of these phyla have a condensed CNS with three primary axon bundles [46], suggestive of an ancestral tripartite subdivision of the CNS. It is true that there are also many examples of species scattered among these phyla that either secondarily lost a condensed polarized CNS or retained a prior ancestral state in which there was only a distributed nervous system. Echinoderms in which *Msx* genes are expressed in the non-neural ectoderm (see above) or the hemichordate *Saccoglossus kowalevskii* which has lost bilateral symmetry to become radially organized [47] may be examples of such derived simplifications of the nervous system. Thus, in our view, the most likely scenario is that the ancestral bilaterian CNS was a condensed nervous system partitioned into at least three DV domains and that loss of centralization has occurred numerous times in different lineages undergoing morphological simplification.

If one assumes a common ancestral origin for BMP-regulation of *msx* genes, one can imagine various scenarios under which BMP-mediated regulation of *msh*/*msx* genes could have switched its effect during evolution. In vertebrates, BMP targets frequently contain *Drosophila* SEs that activate rather than repress transcription. This might be due to Shn proteins losing their repressive activity through changes in the Shn amino acid sequence and/or the lack of components required for repression downstream of Shn. The molecular relatedness of SEs and AEs raises the possibility that ancestral SE-mediated repressive effects on *msh/Msx* expression may have been relatively easy to convert into activating effects in the vertebrate lineage by the loss of the Shn repressor function. Consequently, the increased linker length of zAE could be accounted for by the lack of evolutionary pressure on the SE to meet the sequence requirements for Shn recruitment.

Since the *Drosophila msh* gene is weakly repressed by BMPs (e.g., relative to *ind* and other neural genes such as *AS-C*, *scrt* or *sna* [6]), while vertebrate *msx* genes are weakly activated by BMPs (i.e., high neuroectodermal levels of BMPs are required to activate *msx* genes) an intermediate CRM state may have existed in which BMPs both weakly activated *msx* gene expression within the neuroectoderm at moderate levels while repressing gene expression at the peak BMP levels present in the adjacent epidermis. Indeed the zebrafish *msxB* gene may represent such a bifunctional intermediate condition since *in vivo* studies indicate that high levels of BMPs can inhibit *msxB* expression [12]. It remains to be determined whether such proposed positive and negative inputs are mediated by a single or multiple independent CRM(s). Within different evolutionary lineages such biphasic responses could have then been rendered monophasic in opposing directions to account for the observed differences in the *Drosophila* versus vertebrate or echinoderm *Msx* CRMs. In vertebrates, one potential driving force for reducing the effect of BMP-mediated inhibition may have been the incorporation of BMP expression within the dorsal neural tube itself since this would be expected to generate much higher BMP levels than would result from BMPs diffusing in from the adjacent epidermal ectoderm (e.g., as is the case in *Drosophila*).

In future analyses it will also be important to examine BMP-mediated regulation of additional neural identity genes expressed along the dorsal-ventral axis including the *Gsh* ≈ *ind* and *Nxk2*.2 ≈ *vnd* genes as CRMs controlling expression of each of these genes will have undergone independent evolutionary trajectories. Since there is evidence that laterally and ventrally expressed genes in vertebrates are inhibited by BMPs [48]–[52], and because the more ventrally expressed *ind* gene in *Drosophila* is more sensitive to BMP-mediated repression than *msh* [7], one might expect to find similar, and perhaps conserved ancestral modes, of BMP-mediated repression of these genes across bilateria.

It will also be interesting to understand how flexible the ancestral metazoan state was by investigating the relationship between BMPs and *msx* genes in basal metazoans such as jellyfish. In these diploblastic animals, although the BMP-*msx* relationship has not been tested, BMP2/4 [53] and *msx* [54] homologues are expressed in adjacent regions during development, as is the case in the majority of triploblastic animals.

## Materials and Methods

### Bioinformatics

We identified candidate SE and AE sites in the *msh*, *msxB* and *msx1* CRMs using binding site consensus sequences curated from the literature referenced and used Gene Palette [55]. For this analysis, we used the consensus sequence GNCGNC(N)_5_GNCTG to identify candidate Silencer Elements (SE) and the consensus GGCGCCA(N)_4_GNCV for Activator elements (AE) allowing for single base-pair mismatches to these consensus sequences. We identified candidate zebrafish *msxB* and mouse *Msx1* CRMs by using genome wide alignments for multiple vertebrate species, which indicates regions of high sequence conservation as provided by the UCSC genome browser (http://genome.ucsc.edu).

### CRM-reporter construction and analysis

The 700 bp *msh* CRM is described in Von Ohlen et al., 2009 [25]. All primers used in this study and the corresponding constructs generated can be found in Table S1. The various *Drosophila msh*-CRM constructs were subcloned in pCR-TOPO vectors (Invitrogen) and subsequently cloned into the [P]acman vector [28] as NotI and KpnI restriction fragments. Site-directed mutagenesis PCR methods were adapted from [56]. The primers used to isolate the zebrafish *msxB* CRMs and the mouse *msx1* CRM can be found in Table S1. Zebrafish constructs were cloned into pENTR-TOPO (Invitrogen), transferred to pTol2 by Gateway Recombination and injected in zebrafish embryos as previously described [30].

### Genetic strains and crossing schemes

The *Drosophila dpp* ^h46^ null allele used in this study is Flybase stock number 2061. The 8x HS-dpp stock and its use are described in Biehs *et al* 1996 [6]. The *schnurri* mutant allele is *shn* ^04738^. To generate the *dl dpp st2-dpp^+^* embryos, females that are *Dpdpp*/+; *dl* ^1^ *cn* ^1^ *sca* ^1^/*dpp* ^h46^ *wg* ^sp^ *dl* ^1^ were crossed to *yw*/Y; *dpp* ^h46^ *wg* ^sp^ *st2-dpp* ^+^,*w*+/*CyO* males. The fly strain used to inject all constructs has genotype PBac{yellow[+]-attP-3BVK00002 and injections were outsourced to BestGeneInc (http://www.thebestgene.com/).

The zebrafish strains containing the *hsp70:bmp2b* [57] and *hsp70:chd* [58] transgenes were crossed to stable transgenic lines containing the *msxB*-CRM construct. Embryos at the sphere stage (4hpf) were subjected to heat shock at 37°C for 1 hour and then returned to normal temperature of 28.5°C until they were fixed at the bud – 6 somite (10–11hpf) stage as necessary.

### Electrophoretic mobility shift assays

For electrophoretic mobility shift assays (EMSA), *Drosophila* S2 cells were co-transfected with 50 ng TkvQD and 175 ng Mad- and Med-expression plasmids or with 400 ng of a ShnCT-expression plasmid. Cells were harvested 72 hr after transfection and lysed for 10 min at 4°C in 100 µl of 100 mM Tris (pH 7.5), 1 mM DTT, 0.5% TritonX100 and 1%NP40. Radioactively labeled probes were generated by annealing and filling in partially overlapping oligonucleotides in the presence of [P]-32 ATP. Binding reactions were carried out in a total volume of 25 µl containing 12.5 µl 2x binding buffer (200 mM KCl, 40 mM HEPES (pH 7.9), 40% glycerol, 2 mM DTT, 0.6% BSA and 0.02% NP40), 10000 cpm of radioactively-labeled probe, 1 µl poly dIdC (1 mM) and 7 µl of cleared S2 cell extracts. After incubation for 30 min at 4°C, the reactions were analyzed by non-denaturing 4%polyacrylamide gel electrophoresis followed by autoradiography.

### *In situ* hybridization

Fluorescent *in situ* hybridization methods used were performed according to [59] in *Drosophila* embryos and adapted to zebrafish embryos by increasing the hybridization temperature: 55°C in *Drosophila* to 65°C in zebrafish embryos. Antibodies used: dpERK (Cell Signaling Technology #5683), anti-digoxigenin (Roche), anti-biotin (Roche), Alexa fluor 488, 594, 647 (Invitrogen). We also used colorimetric staining methods performed according to O'Neill and Bier [60]. The DNA template used to generate the *msxB* probe was a generous gift from the Riley lab. Histochemical stain images were acquired using a Nikon optical microscope and fluorescent stain images were collected using a LEICA SP2 confocal microscope. Images were adjusted for color, brightness and contrast using Adobe Photoshop software.

## Supporting Information

Figure S1 Analysis of candidate BMP-responsive and Brk sites in the ME. (A) Diagram of the *Drosophila msh* CRM (ME) indicating the relative position of SEs, AEs and EMSA probes P1-P5. (B) Gel shift assay showing full length Brinker (Brk) and the Brinker DNA binding domain (Brk DNA-BD) bind to control DNA containing a Brk consensus sequence in the presence or absence of Drosophila S2 extracts containing Mad, Medea, and Schnurri (TMMS). The position of probe bound Brinker is indicated by the white arrow, the black arrow indicates the position of Brinker DNA binding domain in complex with the probe. Prior to electrophoresis, the DNA probe was incubated with lysates from *Drosophila* S2 cells transiently expressing Brinker, the Brinker DNA binding domain or constitutively active type I Dpp receptor, Mad, Medea and C-Terminal Schnurri (TMMS). The area boxed in red is a region of the same gel with less developing time. Note that the presence of the pMMS complex does not alter the position of the full-length Brk shift, while the Brk-DNA BD does (i.e., Brk-DNA BD competes with full Brk for binding to that site). (C) Gel shifts induced by pMM and pMMS complexes on oligonucleotides containing candidate BMP-responsive sites. The ability of Brinker to bind several ME regions was also tested. The five different probes, indicated above the gel lanes, were incubated with lysates from *Drosophila* S2 cells transiently expressing Brinker (Brk), constitutively active type I Dpp receptor, Mad, Medea (TMM) and/or C-Terminal Schnurri (S). The white arrow indicates the molecular weight position of probe bound pMMS complexes while the black arrow indicates the position of probe bound pMM complexes on oligonucleotides containing the SE1 (P1) and SE2 (P3) sites. For probes P1 and P3 note that the presence or absence of Brk does not affect the retardation typical of pMMS complexes (lanes 6 and 18) as compared to controls where probes are incubated with TMM and S alone. Compare lanes 3 and 6 for P1 and lanes 15 and 18 for P3. This observation is also noted for probes incubated with extracts containing transiently expressed TMM versus probes incubated with TMM plus Brk, compare lanes 14 and 17 for example. These results suggest that despite the fact that ectopic Brk expression leads to dorsal expansion of *msh* expression [7], Brk does not bind the ME regions surveyed and is not directly regulating the ME via the SE1(P1) or SE2(P3) sites or the other regions surveyed. Regarding probes P4 and P5, no binding signal is detected. (D) Autoradiogram of an EMSA experiment comparing the relative affinities of SE2 and SE2* for pMM and pMMS complexes. The white arrow indicates the molecular weight position of pMMS and the black arrow indicates that of pMM. The labeled oligonucleotide probe corresponding to the SE2* sequence was incubated with *Drosophila* S2 cell lysates transiently expressing constitutively active type 1 Dpp receptor Thick veins, Mad, Medea and C-terminal Schnurri (TMMS) in all lanes except lane 1 (labeled probe alone) and lane 2 (labeled probe with active Tkv, Mad and Med only - TMM). All lanes were loaded with equimolar amounts of labeled SE2* oligonucleotide probe and cell lysate. In lanes 4 to 11, progressively greater amounts of unlabeled SE* probe were added to the mix, while in lanes 13 to 20, increasing amounts of unlabeled SE2 oligonucleotide probe was added. In these experiments unlabeled oligonucleotides act as competitors for pMMS complexes against the labeled probe. The decrease in the amount of labeled probe shifted as the competitor concentration increases is more pronounced with the unlabeled SE2* competitor than with the unlabeled SE2 competitor revealing that the wild-type SE2 probe is less effective as a competitor than the SE2* probe. Probe sequences are indicated below the gel. Bold bases indicate the GC-rich and SBE sites, lower case indicates the mutated base that differentiates SE2* from SE2.
(TIF)

Figure S2 Different Dpp doses do not elicit *msh* expression and changes in EGF signaling do not affect the dorsal border of the *msh* expression domain. (A,B) Early stage *Drosophila* embryos with varying genetic dosages of *dpp*. In situ hybridization of embryos oriented with anterior regions to the left in both images. (A) Embryos lacking Dorsal but heterozygous for *dpp*, retain slight *msh* expression in head regions and strong *msh* expression in tail regions but *msh* is absent from middle regions. (B) To approximate a situation where the Dpp dose is in between the wild-type and heterozygous conditions, we added the *eve-stripe-2-dpp+* construct to an embryo lacking maternal Dorsal and zygotically heterozygous for *dpp*. In this particular genetic background, *msh* expression is severely reduced as well. These results reinforce the idea that Dpp does not have an activating role in *msh* regulation in the absence of Dorsal signaling in *Drosophila melanogaster* at these stages. (C) Ventro-lateral view of a wild-type embryo (this and all other embryos with anterior to the left), depicting the expression of activated ERK (detected with an anti-dpERK antibody - yellow) relative to *ind* mRNA (red) and *msh* mRNA (green). Note that dpERK staining is not detected dorsal to the *ind* expression domain. (D) Dorso-lateral view of an embryo, anterior to the left, ectopically expressing a secreted form of Spitz (s-Spitz) under the control of a *Kruppel* (*Kr*) driver using the GAL4/UAS system. Ectopic expression of s-Spitz leads to a localized dorsal expansion (white arrows) of *ind* (red) within the *Kruppel* domain (detected by *gal4* mRNA - yellow) while *msh* (green) expression is unaffected. (E) Dorso-lateral view of a *cic* mutant embryo. *ind* (red) expression expands dorsally as previously reported [63], while the *msh* (green) domain loses some vent ral expression (presumably due to repression by Ind) but its dorsal border remains unaffected.
(TIF)

Figure S3 Characterization of BMP responsive sites in the *msxB*-CRM. (A) Gel shift assay identifying a single site in the zebrafish 671 bp minimal *msxB* CRM that binds to *Drosophila* pMad and Medea. A diagram with the relative position of the probes within the *msxB*-CRM as well as a conservation map of the *msxB*-CRM region among selected species is shown. Labeled oligonucleotide probes corresponding to different candidate regions containing AE-related sites of the *msxB*-CRM were incubated with extracts from *Drosophila* S2 cells over-expressing activated Tkv (to induce BMP signaling), Med and Mad (TMM). The black arrow indicates the position of probe bound pMM complexes. Only probe Msx11 (lanes 1 and 2 on the gel) show a BMP-dependent shift at the pMM molecular weight position. This probe spans the most highly conserved region of the *msxB*-CRM in mammals and fish. (B) Map of the zAE region corresponding to the Msx11 probe within *msxB*-CRM. The relative position of Msx11A-F oligonucleotide probes and putative AE sites are represented. Gel shift assay demonstrating that pMM complexes can assemble within the conserved region of the *msxB*-CRM. Prior to electrophoresis, the DNA probes were incubated with lysates from *Drosophila* S2 cells transiently expressing Mad and Medea (MM), constitutively active type I Dpp receptor, Mad and Medea (TMM) or constitutively active type I Dpp receptor, Mad, Medea and C-Terminal Schnurri (TMMS). The white arrow indicates the molecular weight position of pMMS and the black arrow indicates the molecular weight position of pMM. As a positive control, the P3 probe corresponding to the SE2 region of the *Drosophila msh*-CRM (Fig. S1) was used. The Msx11 oligonucleotide is capable of assembling pMM complexes and these seem unaffected by the presence of Shn. To narrow down the binding sites of Mad and Med, sub-regions of the Msx11 probe labeled Msx11A-F were incubated with TMM lysates. Msx11F, which is contained in Msx11A, represents the minimal shifted probe, indicating that pMad/Med complexes are assembling within this sub-region. (C) Mutant versions of Msx11F probe were incubated with *Drosophila* S2 cell TMM lysates and gel shift assays were performed. Mutating the entire predicted 16 bp linker region (lm) did not affect the ability to assemble pMM complexes in vitro. When the GC-rich sequence is mutated at either the 5′ or 3′ end (GCR1 and GCR2, respectively) probe retardation was no longer observed. Changing only the predicted SBE site severely reduced the shifted probe signal. These results suggest complexes with the usual 2 Mad to 1 Med ratio are assembling on the Msx11F probe [9]. In addition, the linker length does not seem to be a factor in the formation of pMM complexes since changing it to 15 (l15), 12 (l12), 8 (l8), 6 (l6) or 2 (l2) base pairs did not prevent the probe from being shifted as long as the GC-rich and SBE regions remained intact. (D) Gel shift assay demonstrating that the presence of Shn does not affect the formation of pMM complexes on Msx11F *in vitro*. Interestingly, however, Shn can bind together with Mad/Med if the linker length is reduced to 5 base pairs (l5).
(TIF)

Table S1 Primers used in this study.
(DOCX)
