# Supplementary figures and images for: BMPs Regulate msx Gene Expression in the Dorsal Neuroectoderm of Drosophila and Vertebrates by Distinct Mechanisms
Source: PLoS Genet. 2014 Sep 11;10(9):e1004625. doi: 10.1371/journal.pgen.1004625 (PMC4161316; doi:10.1371/journal.pgen.1004625)

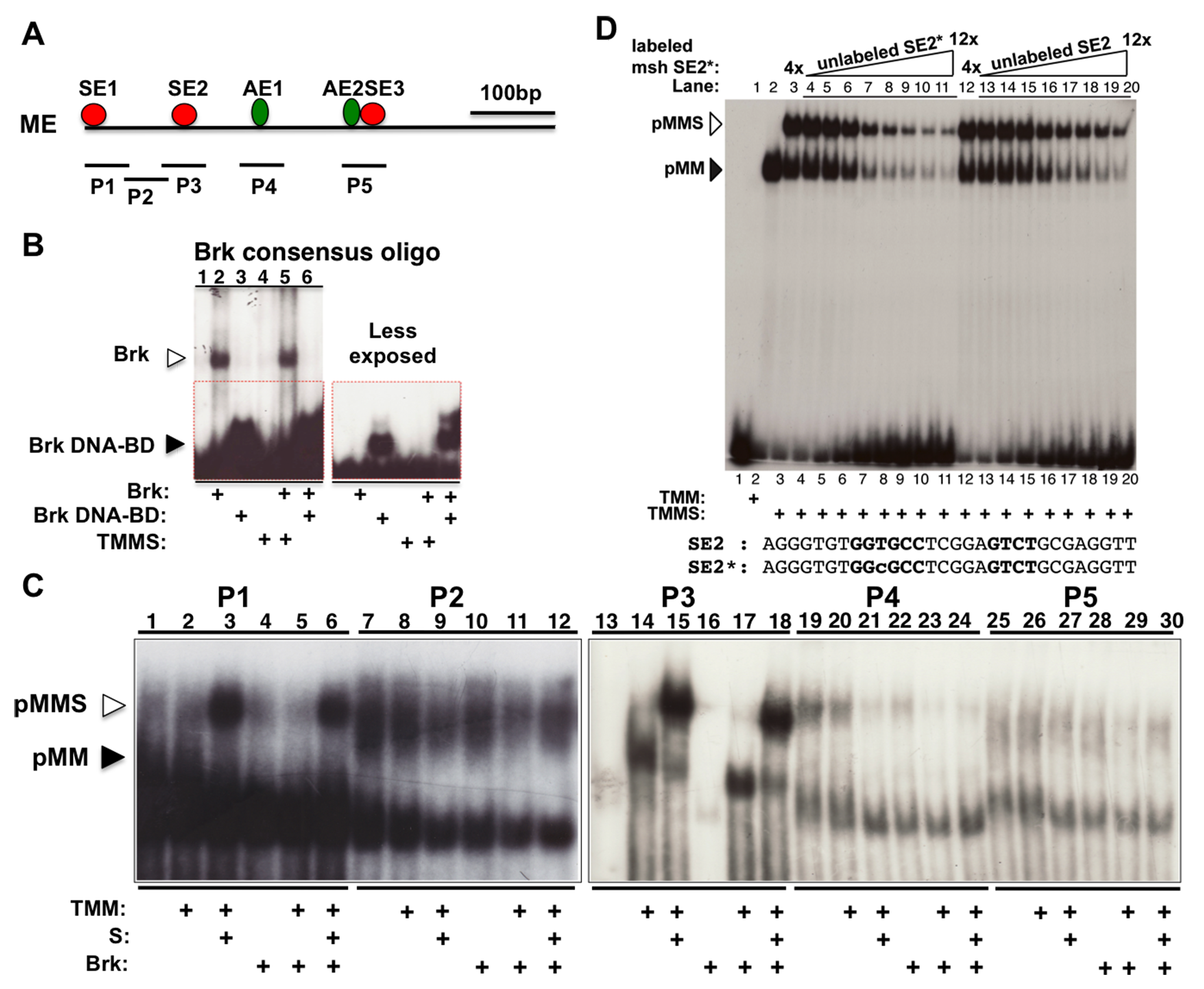

Supplement: Figure S1 — Analysis of candidate BMP-responsive and Brk sites in the ME. (A) Diagram of the Drosophila msh CRM (ME) indicating the relative position of SEs, AEs and EMSA probes P1-P5. (B) Gel shift assay showing full length Brinker (Brk) and the Brinker DNA binding domain (Brk DNA-BD) bind to control DNA containing a Brk consensus sequence in the presence or absence of Drosophila S2 extracts containing Mad, Medea, and Schnurri (TMMS). The position of probe bound Brinker is indicated by the white arrow, the black arrow indicates the position of Brinker DNA binding domain in complex with the probe. Prior to electrophoresis, the DNA probe was incubated with lysates from Drosophila S2 cells transiently expressing Brinker, the Brinker DNA binding domain or constitutively active type I Dpp receptor, Mad, Medea and C-Terminal Schnurri (TMMS). The area boxed in red is a region of the same gel with less developing time. Note that the presence of the pMMS complex does not alter the position of the full-length Brk shift, while the Brk-DNA BD does (i.e., Brk-DNA BD competes with full Brk for binding to that site). (C) Gel shifts induced by pMM and pMMS complexes on oligonucleotides containing candidate BMP-responsive sites. The ability of Brinker to bind several ME regions was also tested. The five different probes, indicated above the gel lanes, were incubated with lysates from Drosophila S2 cells transiently expressing Brinker (Brk), constitutively active type I Dpp receptor, Mad, Medea (TMM) and/or C-Terminal Schnurri (S). The white arrow indicates the molecular weight position of probe bound pMMS complexes while the black arrow indicates the position of probe bound pMM complexes on oligonucleotides containing the SE1 (P1) and SE2 (P3) sites. For probes P1 and P3 note that the presence or absence of Brk does not affect the retardation typical of pMMS complexes (lanes 6 and 18) as compared to controls where probes are incubated with TMM and S alone. Compare lanes 3 and 6 for P1 and lane [file pgen.1004625.s001.tif]

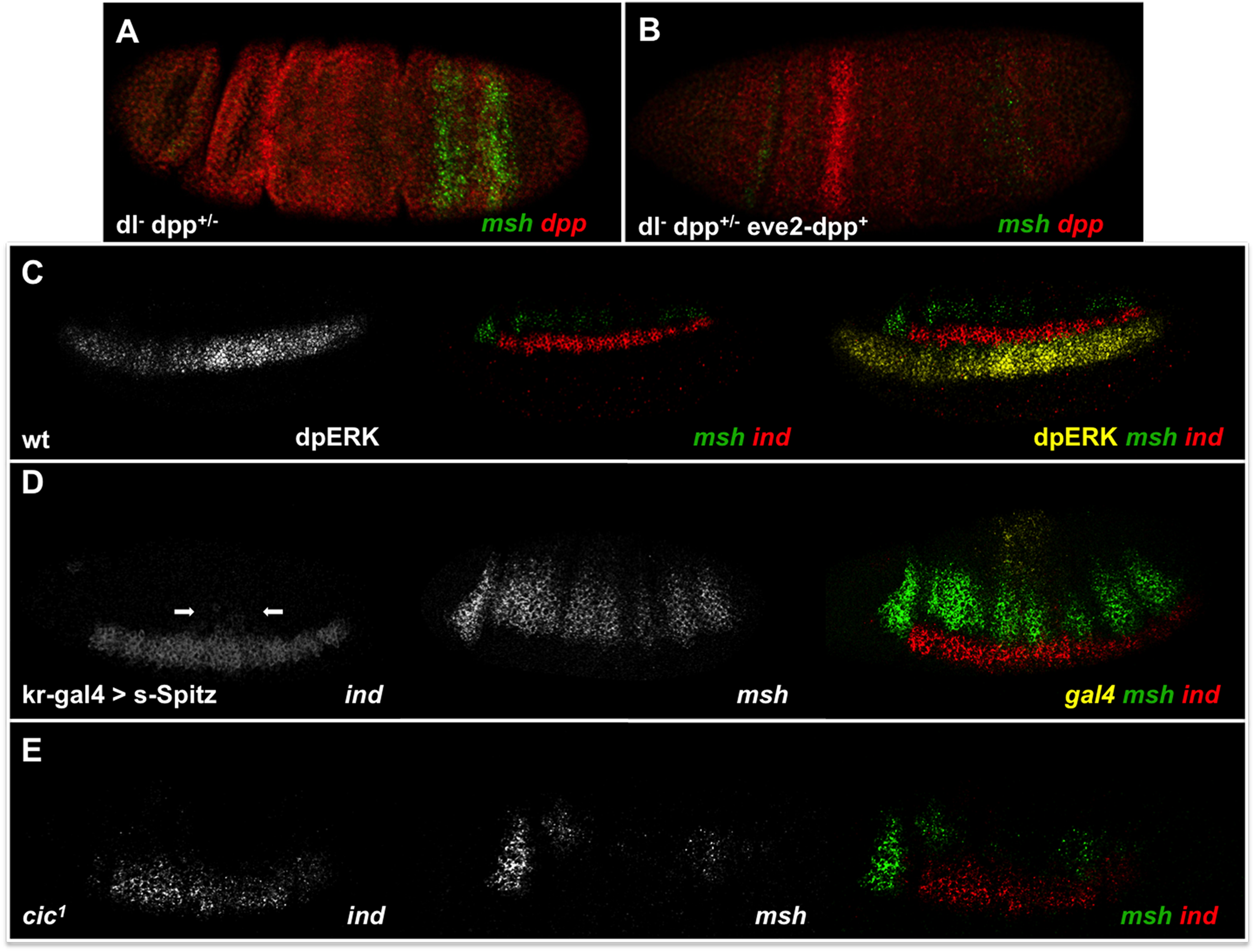

Supplement: Figure S2 — Different Dpp doses do not elicit msh expression and changes in EGF signaling do not affect the dorsal border of the msh expression domain. (A,B) Early stage Drosophila embryos with varying genetic dosages of dpp. In situ hybridization of embryos oriented with anterior regions to the left in both images. (A) Embryos lacking Dorsal but heterozygous for dpp, retain slight msh expression in head regions and strong msh expression in tail regions but msh is absent from middle regions. (B) To approximate a situation where the Dpp dose is in between the wild-type and heterozygous conditions, we added the eve-stripe-2-dpp+ construct to an embryo lacking maternal Dorsal and zygotically heterozygous for dpp. In this particular genetic background, msh expression is severely reduced as well. These results reinforce the idea that Dpp does not have an activating role in msh regulation in the absence of Dorsal signaling in Drosophila melanogaster at these stages. (C) Ventro-lateral view of a wild-type embryo (this and all other embryos with anterior to the left), depicting the expression of activated ERK (detected with an anti-dpERK antibody - yellow) relative to ind mRNA (red) and msh mRNA (green). Note that dpERK staining is not detected dorsal to the ind expression domain. (D) Dorso-lateral view of an embryo, anterior to the left, ectopically expressing a secreted form of Spitz (s-Spitz) under the control of a Kruppel (Kr) driver using the GAL4/UAS system. Ectopic expression of s-Spitz leads to a localized dorsal expansion (white arrows) of ind (red) within the Kruppel domain (detected by gal4 mRNA - yellow) while msh (green) expression is unaffected. (E) Dorso-lateral view of a cic mutant embryo. ind (red) expression expands dorsally as previously reported [63], while the msh (green) domain loses some vent ral expression (presumably due to repression by Ind) but its dorsal border remains unaffected. (TIF) [file pgen.1004625.s002.tif]

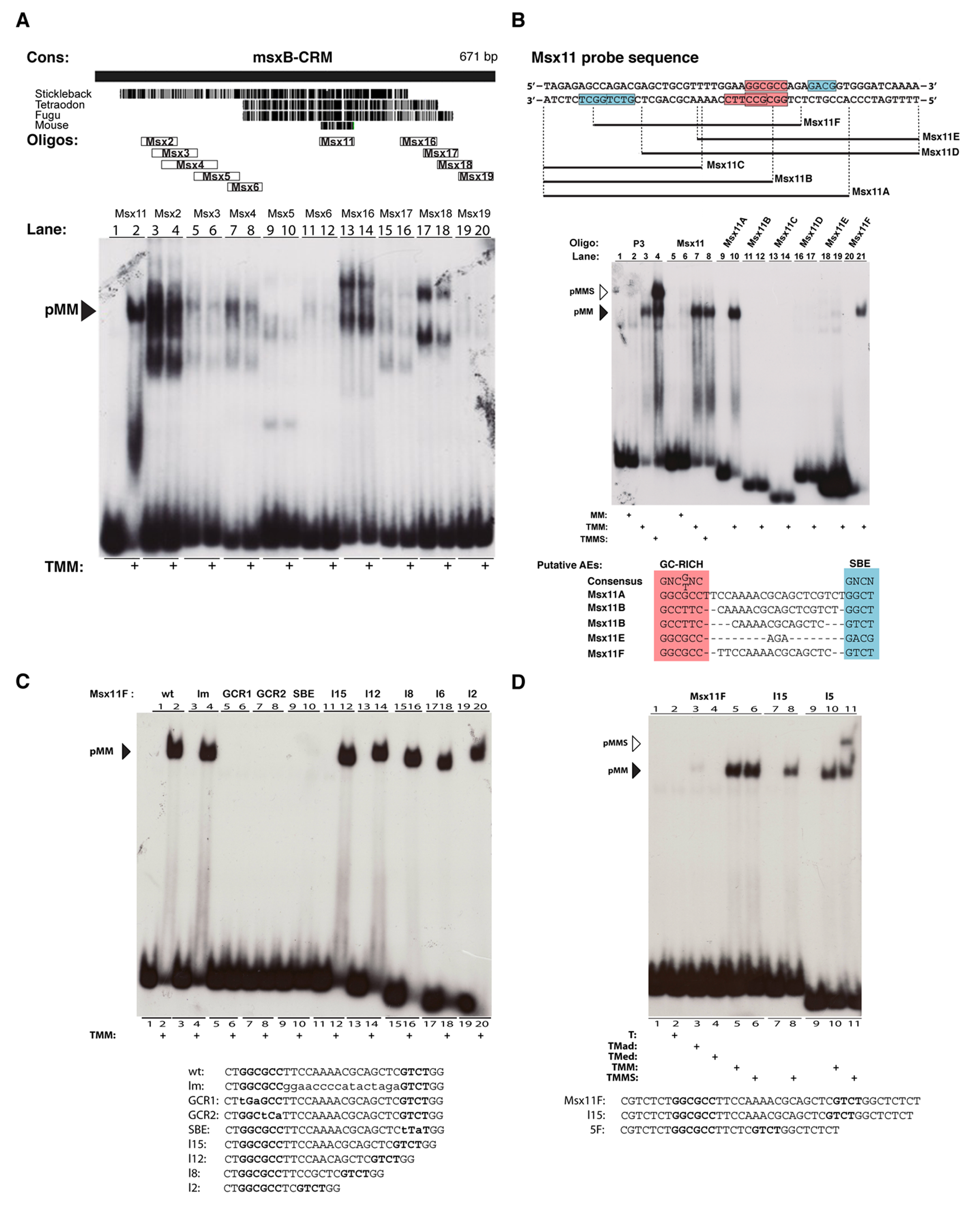

Supplement: Figure S3 — Characterization of BMP responsive sites in the msxB-CRM. (A) Gel shift assay identifying a single site in the zebrafish 671 bp minimal msxB CRM that binds to Drosophila pMad and Medea. A diagram with the relative position of the probes within the msxB-CRM as well as a conservation map of the msxB-CRM region among selected species is shown. Labeled oligonucleotide probes corresponding to different candidate regions containing AE-related sites of the msxB-CRM were incubated with extracts from Drosophila S2 cells over-expressing activated Tkv (to induce BMP signaling), Med and Mad (TMM). The black arrow indicates the position of probe bound pMM complexes. Only probe Msx11 (lanes 1 and 2 on the gel) show a BMP-dependent shift at the pMM molecular weight position. This probe spans the most highly conserved region of the msxB-CRM in mammals and fish. (B) Map of the zAE region corresponding to the Msx11 probe within msxB-CRM. The relative position of Msx11A-F oligonucleotide probes and putative AE sites are represented. Gel shift assay demonstrating that pMM complexes can assemble within the conserved region of the msxB-CRM. Prior to electrophoresis, the DNA probes were incubated with lysates from Drosophila S2 cells transiently expressing Mad and Medea (MM), constitutively active type I Dpp receptor, Mad and Medea (TMM) or constitutively active type I Dpp receptor, Mad, Medea and C-Terminal Schnurri (TMMS). The white arrow indicates the molecular weight position of pMMS and the black arrow indicates the molecular weight position of pMM. As a positive control, the P3 probe corresponding to the SE2 region of the Drosophila msh-CRM (Fig. S1) was used. The Msx11 oligonucleotide is capable of assembling pMM complexes and these seem unaffected by the presence of Shn. To narrow down the binding sites of Mad and Med, sub-regions of the Msx11 probe labeled Msx11A-F were incubated with TMM lysates. Msx11F, which is contained in Msx11A, represents the minimal shifted probe, indicat [file pgen.1004625.s003.tif]
