# Supplementary material for: BMPs Regulate msx Gene Expression in the Dorsal Neuroectoderm of Drosophila and Vertebrates by Distinct Mechanisms
Source: PLoS Genet. 2014 Sep 11;10(9):e1004625. doi: 10.1371/journal.pgen.1004625 (PMC4161316; doi:10.1371/journal.pgen.1004625)
Supplement: Table S1 — Primers used in this study. (DOCX) [file pgen.1004625.s004.docx]

**Table S1.** Primers used in this study.

| **Construct Name** | **DNA Templates** | **Primer Pair (5’->3’)** |
| --- | --- | --- |
| *msh*-CRM | BDGP *w^-^* genomic DNA | msh1- GTTGCCCCGAGGTCTGGAGTTAGT  msh2- GCAGCTGAAGCTGGAAACTGAA |
| D1 | BDGP *w^-^* genomic DNA | D1F- GCTTTTGGGCTAAGCTCACAGCAGG  msh2 |
| SE1 | pTopo-mshCRM | SE1f- TGTGCTGGAATTCGCCCTTAACCATTGAGCCACTCAGAGTTAGTTTCTGGCATTCT  SE1R- AGAATGCCAGAAACTAACTCTGAGTGGCTCAATGGTTAAGGGCGAATTCCAGCACA |
| SE2 | pTopo-mshCRM | SE2f- CACCACCAGGGTGTAACATTTCGGAACTCACGAGGTTTGCTTTTGGG  SE2r- CCCAAAAGCAAACCTCGTGAGTTCCGAAATGTTACACCCTGGTGGTG |
| SE1SE2 | pTopo-SE1 | SE2f  SE2r |
| SE2* | pTopo-mshCRM | SE2*f- CCAGGGTGTGGCGCCTCGGAGTCTGCGAGGTTTGCTTTTGGG  SE2*r- CCCAAAAGCAAACCTCGCAGACTCCGAGGCGCCACACCCTGG |
| D2 | pTopo-mshCRM | D2F- CTAAGCTCACAGCAGGATTGCACTATGTGTCCTCAGGG  D2R- GGACACATAGTGCAATCCTGCTGTGAGCTTAGCCC |
| D3 | pTopo-mshCRM | D3F- GCACTATGTGTCCTCAGGGCAGGATTCGAGTGCTTGTGG  D3R- CCACAAGCACTCGAATCCTGCTGAGGACACATAGTGCAATCC |
| D4 | BDGP *w^-^* genomic DNA | msh1  D4r- ACAAGCACTCGAATCCTGCC |
| AE2 | pTopo-mshCRM | AE2f-GGGACACCCTACCTGCGTATGATTAACTCGATAATCTGTGGGTGATGCC  AE2r-GGCATCACCCACAGATTATCGAGTTAATCATACGCAGGTAGGGTGTCCC |
| SE1SE2AE2 | pTopo-SE1SE2 | AE2f  AE2r |
| *msxB*-CRM  (2431bb) | Zebrafish genomic DNA | mBf- CAACAACTTCTAACACACTCCGTCCC  mBr- CTTGGCCAGTTTGATTTGATACCCGC |
| *Msx1*-CRM | Black 6 mice genomic DNA | msxE1- AACTTGTTAATAAGGCAAGGCCAGCGTGGC  msxE2- GGCTGCTCTTGGGAATGAAAAAGGCCAGGC |
| *msxB*-CRM (671bp) | Zebrafish genomic DNA | mB1f- GCGACCAGAAAAGTGTAATTATAGGGCTCG  mB3r- CCCTGTAGTTCACTGGATTAAACTTGG |
